# The role of the lateral hypothalamus and orexin in ingestive behavior: a model for the translation of past experience and sensed deficits into motivated behaviors

**DOI:** 10.3389/fnsys.2014.00216

**Published:** 2014-11-13

**Authors:** Seth W. Hurley, Alan Kim Johnson

**Affiliations:** ^1^Department of Psychology, University of IowaIowa City, IA, USA; ^2^Department of Pharmacology, University of IowaIowa City, IA, USA; ^3^Department of Health and Human Physiology, University of IowaIowa City, IA, USA; ^4^François M. Abboud Cardiovascular Center, University of IowaIowa City, IA, USA

**Keywords:** motivation, homeostasis, lateral hypothalamus, neural plasticity, salt appetite, thirst, food intake, orexin

## Abstract

The hypothalamus has been recognized for its involvement in both maintaining homeostasis and mediating motivated behaviors. The present article discusses a region of the hypothalamus known as the lateral hypothalamic area (LHA). It is proposed that brain nuclei within the LHA including the dorsal region of the lateral hypothalamus (LHAd) and perifornical area (PeF) provide a link between neural systems that regulate homeostasis and those that mediate appetitive motivated behaviors. Functional and immunohistochemical data indicate that the LHA promotes many motivated behaviors including food intake, water intake, salt intake, and sexual behavior. Anatomical tracing experiments demonstrate that the LHA is positioned to receive inputs from brain areas involved in regulating body fluid and energy homeostasis. Regions within the LHA send dense projections to the ventral tegmental area (VTA), providing a pathway for the LHA to influence dopaminergic systems generally recognized to be involved in motivated behaviors and their reinforcement. Furthermore, the LHA contains neurons that synthesize orexin/hypocretin, a neuropeptide that promotes many appetitive motivated behaviors. The LHA also receives inputs from brain areas involved in reward-related learning and orexin neuron activation can become conditioned to environmental stimuli that are associated with rewards. Therefore, it is hypothesized that the LHA integrates signaling from areas that regulate body fluid and energy balance and reward-related learning. In turn, this information is “fed into” mesolimbic circuitry to influence the performance of motivated behaviors. This hypothesis may foster experiments that will result in an improved understanding of LHA function. An improved understanding of LHA function may aid in treating disorders that are associated with an excess or impairment in the expression of ingestive behavior including obesity, anorexia, impairments in thirst, salt gluttony, and salt deficiency.

## Introduction

In order to survive animals must maintain energy and fluid homeostasis. Calories are continually lost through processes that maintain basic life functions and as a result of behaving. Similarly, terrestrial animals continually lose water and sodium to the environment due to normal physiological and environmental processes including respiration, transpiration, perspiration, urination, and defecation. Some less-common circumstances pose a significant threat to both energy and body fluid homeostasis. For example, sicknesses can evoke states of hypophagia coupled with diarrhea and vomiting that deplete the body of water and sodium. Once the body has become deficient in calories, water, or sodium it is critical for an animal to seek out and ingest substances in the environment to restore homeostasis.

The central nervous system generates motivational states to promote the seeking and ingestion of substances in the environment. The motivational state of hunger, or the seeking and ingestion of food, is necessary for an animal to restore deficits in energy homeostasis. Thirst and sodium appetite (also known as [AKA] salt appetite), or the acquisition and consumption of water and sodium, are necessary to restore fluid balance. These motivated states are accompanied by central nervous system processes that energize behavior (i.e., produce a state of psychological arousal and encourage locomotor behavior) and promote goal directed behavior (Bindra, 1959; Bolles, 1975). It has been demonstrated that many motivational states are accompanied by a hedonic shift where the pleasurable or aversive responses evoked by specific stimuli are enhanced or inhibited (Garcia et al., 1974; Fanselow and Birk, 1982; Berridge et al., 1984; Mehiel and Bolles, 1988; Berridge and Schulkin, 1989). For example, when sodium-replete rats receive intra-oral infusions of hypertonic saline solutions they exhibit species-specific behavioral responses indicative of aversion (Grill and Norgren, 1978; Berridge et al., 1984) and sodium-replete rats will generally eschew hypertonic saline solutions (Robinson and Berridge, 2013). However, when rats become sodium deficient they exhibit approach behaviors towards saline solutions and will perform responses that are instrumental to obtain sodium (AKA operant responses; Berridge et al., 1984; Clark and Bernstein, 2006; Robinson and Berridge, 2013). Under sodium deficient conditions they even display behavioral responses indicative of pleasure rather than aversion when hypertonic saline solutions are infused intra-orally (Berridge et al., 1984). Similarly, people rate salty foods as more palatable when they are sodium deficient (McCance, 1936; Beauchamp et al., 1990).

The motivated states of hunger, thirst, and salt appetite are strongly influenced by an animal’s current state of energy and fluid balance (i.e., homeostatic state). It is reasonable to conceptualize the neural apparatus that monitor energy and fluid homeostasis as sensory systems in their own right. With respect to energy balance, the arcuate nucleus of the hypothalamus (ARH) has received significant attention for its role in sensing peripheral signals related to hunger and satiety (Schwartz et al., 2000). An ensemble of forebrain nuclei lying along the lamina terminalis (LT) are important for detecting signals related to body fluid status (Denton et al., 1996; Johnson and Thunhorst, 1997). The specific structures along the LT are the subfornical organ (SFO), median preoptic area (MnPO), and organum vasculosum of the lamina terminalis (OVLT). To facilitate exposition these structures are collectively referred to as the LT. The SFO and OVLT are sensory circumventricular organs, or brain areas that lack a true blood-brain barrier (Johnson and Gross, 1993) which allows them to monitor substances in the blood that act as indicators of body fluid status (Johnson and Thunhorst, 1997, 2007). It is also worth noting here that there is currently debate as to whether the ARH lacks a blood-brain barrier and is a true circumventricular organ (Mimee et al., 2013). As such, it has been proposed that the LT may also function to detect signals related to energy balance (Mimee et al., 2013; Smith and Ferguson, 2014).

It is important to realize that the ingestion of food, water, and sodium require coordinated activity between neural circuits that sense energy and fluid status and the neural circuitry involved in mobilizing motivated behaviors (Garcia et al., 1974; Roitman et al., 1997; Kelley and Berridge, 2002; Liedtke et al., 2011). Therefore, the brain areas that monitor fluid and energy status must be able to project onto the areas that regulate motivation and reward. One final common pathway that is implicated in the generation of all appetitive motivated behaviors investigated thus far is the dopaminergic projection from the ventral tegmental area (VTA) to the nucleus accumbens (AKA the mesolimbic dopamine system and the A10 dopaminergic cell group; Mogenson et al., 1980; Bozarth, 1994). The ARH and the LT, which are involved in sensing energy and fluid balance, do not appear to directly innervate the VTA (Phillipson, 1979; Geisler and Zahm, 2005; however the ARH does directly project to the nucleus accumbens; Yi et al., 2006; van den Heuvel et al., 2014). As there are no direct projections to the VTA it is likely that areas in the hypothalamus may aid in “bridging the gap” between homeostasis and motivation and reward systems (Mogenson et al., 1980; Swanson and Mogenson, 1981; Swanson and Lind, 1986). For example, retrograde tracing studies have shown that a large region of the hypothalamus contains neurons that project to the VTA (Geisler and Zahm, 2005). This region extends from the dorsomedial hypothalamus (DMH) to the dorsal region of the lateral hypothalamus (LHAd) and appears to be present across the entire anterior-posterior extent of the hypothalamus.

## The evidence supporting a role for the LHA in integrating homeostatic state with motivation and reward systems

In a classic paper, Stellar (1954) proposed a hypothalamus-centered theory of motivation. Stellar theorized that the hypothalamus contained anatomically dissociable “centers” and each center played a critical role in the promotion of specific motivated behaviors. For example, he posited that the hypothalamus contained centers that specifically control sex, satiety, hunger, and sleep. Stellar’s proposal received careful experimental scrutiny and was found to be inadequate to explain emerging data (Miller et al., 1964; Miller, 1965; Hoebel and Teitelbaum, 1966; Booth et al., 1969). Despite the fact that Stellar’s theory fell short of elucidating the role of the hypothalamus in specific motivated behaviors, considerable evidence now suggests that areas within the hypothalamus do, in fact, play an important role in promoting appetitive motivated behaviors in general.

Classic experiments that employed short bursts of electrical stimulation directed at the lateral hypothalamic area (LHA) demonstrated that the LHA is involved in motivation and reward processes. Olds and Milner (1954) originally found that rats will perform an operant to obtain acute electrical stimulation of the LHA, an experimental paradigm sometimes referred to as self-stimulation or brain stimulation reward. They interpreted their finding to mean that electrical stimulation of the LHA was rewarding (i.e., LHA stimulation evoked a subjective state of pleasure). If this assessment is correct, it would suggest that some neurons within the LHA are functionally important for coding pleasure from the consumption of rewards. However, others have suggested LHA stimulation may actually produce a subjective state of craving rather than pleasure *per se* (Berridge and Valenstein, 1991). If this interpretation is true it would suggest that a subset of neurons located in the LHAd are involved in the craving that drives animals to seek rewards. It is likely that the motivational and rewarding properties of LHA stimulation are the result of the activation of neurons in the LHA that project to the mesolimbic dopamine system (Phillipson, 1979; Geisler and Zahm, 2005). Recent experiments utilizing anatomical mapping of “hedonic hotspots”, or brain areas that appear to code pleasure (Peciña and Berridge, 2000), show that neurons located in the anterior LHA project to a hedonic hotspot in the dorsomedial nucleus accumbens shell (Thompson and Swanson, 2010), and it is possible that LHA stimulation may activate these projection neurons to evoke a sensation of pleasure. Interestingly, if the LHA is stimulated for sufficient intervals (~10–30 s) rats will perform motivated behaviors including drinking, eating, and copulatory behaviors (Wise, 1968). Furthermore, lesions of the LHA abolish food and water intake, copulation, and impair or abolish sodium appetite (Anand and Brobeck, 1951; Montemurro and Stevenson, 1957; Teitelbaum and Epstein, 1962; Wolf, 1964; Wolf and Quartermain, 1967; Cagguila et al., 1973; Grossman et al., 1978; Hansen et al., 1982).

Disruptions in energy or fluid balance alter responding for self-stimulation (Olds, 1958; Morris et al., 2006, 2010). Olds (1958) originally found that food-depriving rats and inducing the motivational state of hunger increased responding for self-stimulation. Furthermore, increased responding for self-stimulation during food deprivation can be prevented by administration of leptin, a hormone that promotes satiety (Fulton et al., 2000). In contrast to food deprivation, sodium depletion reduces responding for self-stimulation (Morris et al., 2010). Reduced responding for self-stimulation is even observed when rats are made salt hungry through administration of an exogenous hormone that promotes salt appetite; despite the fact rats maintain sodium balance during this treatment (Morris et al., 2006). It is unclear as to why the motivational states of hunger and salt appetite produce opposite effects on self-stimulation. However, these studies demonstrate that hunger and salt appetite alter self-stimulation responding and this effect appears to be independent of actual disruptions in energy or fluid homeostasis. For example, leptin normalizes self-stimulation responding without correcting lost calories (Fulton et al., 2000) and self-stimulation responding can be decreased through manipulations that evoke salt hunger without actually inducing a sodium deficit (Morris et al., 2006). Importantly, these experiments support the present hypothesis by showing that the LHA is sensitive to an animal’s motivational state.

**Figure 1:**
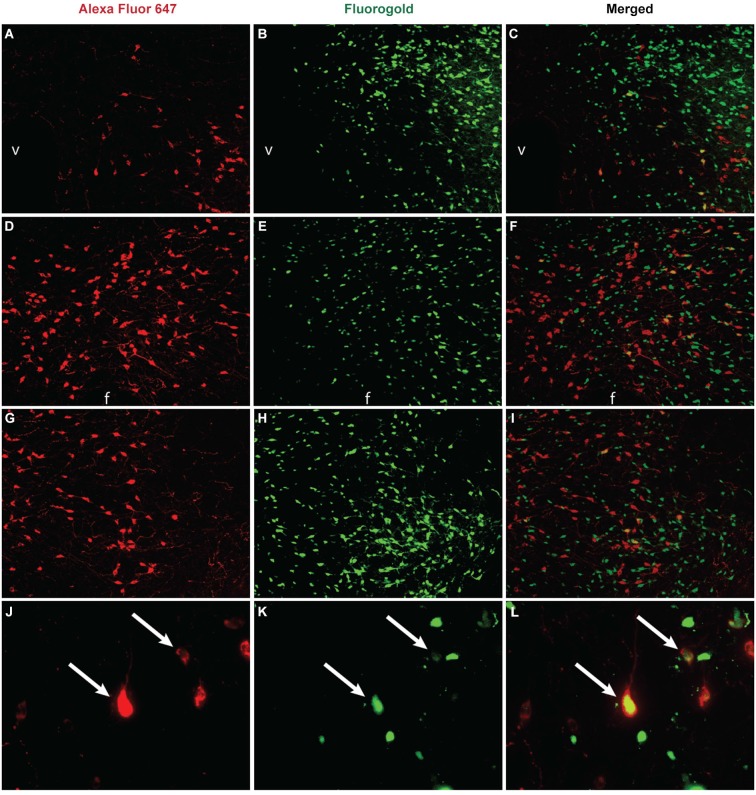
**Unpublished data from authors**. Co-labeling between orexin and VTA projection neurons in the hypothalamus. The retrograde tracer Fluoro-Gold (Fluorochrome, Denver CO) was microinjected (2% in 250 nl) into the VTA, brains were collected and sliced at 40µm, and then tissue was stained for orexin A immunoreactivity via overnight incubation with an anti-orexin A antibody (1:8000, Phoenix Pharmaceuticals, Burlingame CA) and visualized through incubation with Alexa Fluor 647 (1:200, Jackson ImmunoResearch, West Grove PA) for 1 h. Images **(A–I)** were taken at 10x magnification and images **(J–L)** were taken at 40x. In the DMH **(A–C)**, significant orexin neuron labeling was observed **(A)** in addition to retrogradely labeled neurons from the VTA **(B)**, and some orexin neurons projected to the VTA (**C**, yellow labeling). In the PeF **(D–F)** a subset of orexin neurons **(D)** and retrogradely labeled neurons from the VTA **(E)** co-localized **(F)**. In the LHAd **(G–I)** a subset of orexin neurons **(G)** and retrogradely neurons from the VTA **(H)** colocalized **(I)**. A 40x magnification of labeling observed in the DMH is presented in panels **(J–L)**. Double-labeled neurons are indicated by white arrows. v = ventricle, f = fornix.

Some of the strongest evidence supporting a role for the hypothalamus in promoting motivated behavior comes from studies examining orexin (AKA hypocretin). Orexin is a neuropeptide that is expressed primarily in the caudal half of the hypothalamus where it is distributed in an arc that extends from the DMH to the LHAd (Figure 1). Orexin appears to be the only known centralized peptide neurotransmitter system as orexin neurons from a relatively circumscribed area send distal projections to diverse brain areas (Peyron et al., 1998). Functionally, orexin neurons have been heavily implicated in a variety of motivated behaviors (Harris et al., 2005; Borgland et al., 2009). Orexin has received significant attention for its capacity to elicit robust food intake (hence the name orexin; Sakurai et al., 1998; Choi et al., 2010) but it is also involved in promoting thirst, salt appetite (Kunii et al., 1999; Hurley et al., 2013a), and reproductive behavior (Muschamp et al., 2007; Di Sebastiano et al., 2010). Orexin neurons can be putatively organized into three cell-clusters in the hypothalamus: a cluster in the DMH, perifornical area (PeF), and the LHAd (Figure 1). Both the PeF and LHAd are regions located in the LHA while the DMH lies medially where it abuts the third ventricle. Each orexin cell-cluster contains a subset of orexin neurons that project to the VTA (Figure 1; Fadel and Deutch, 2002), and orexin is capable of depolarizing neurons in the VTA (Korotkova et al., 2003). Therefore, orexin neurons provide a mechanism for areas within the LHA to tap into systems traditionally conceived of as being involved in motivation and reward. Evidence also indicates that orexin neurons have direct projections to the nucleus accumbens shell (Peyron et al., 1998; Kampe et al., 2009) where they may act to promote motivated behaviors (Thorpe and Kotz, 2005).

Other major functions of orexin include promoting arousal (Hagan et al., 1999) and sympathetic nervous system responses including the elevation of blood pressure (Samson et al., 1999; Ferguson and Samson, 2003; Kayaba et al., 2003) and the release of stress hormones (Kuru et al., 2000; Spinazzi et al., 2006). It is likely that orexin neurons are activated while an animal is experiencing caloric, hydrational, or sodium deficiency or is in a state of sexual arousal. The subsequent release of orexin throughout the neuraxis encourages the performance of goal-directed behavior by activating brain systems involved in promoting arousal, attention, sympathetic activity, and motivated behavior. Sympathetic activation supports energy mobilization (e.g., increased blood pressure and available glucose levels, and stress hormone release) as well as the redistribution of blood necessary to support increased locomotor activity. Together these central and peripheral responses serve to increase the likelihood that an animal will successfully seek out and consume environmental reinforcers that restore energy and hydrational homeostasis.

**Figure 2:**
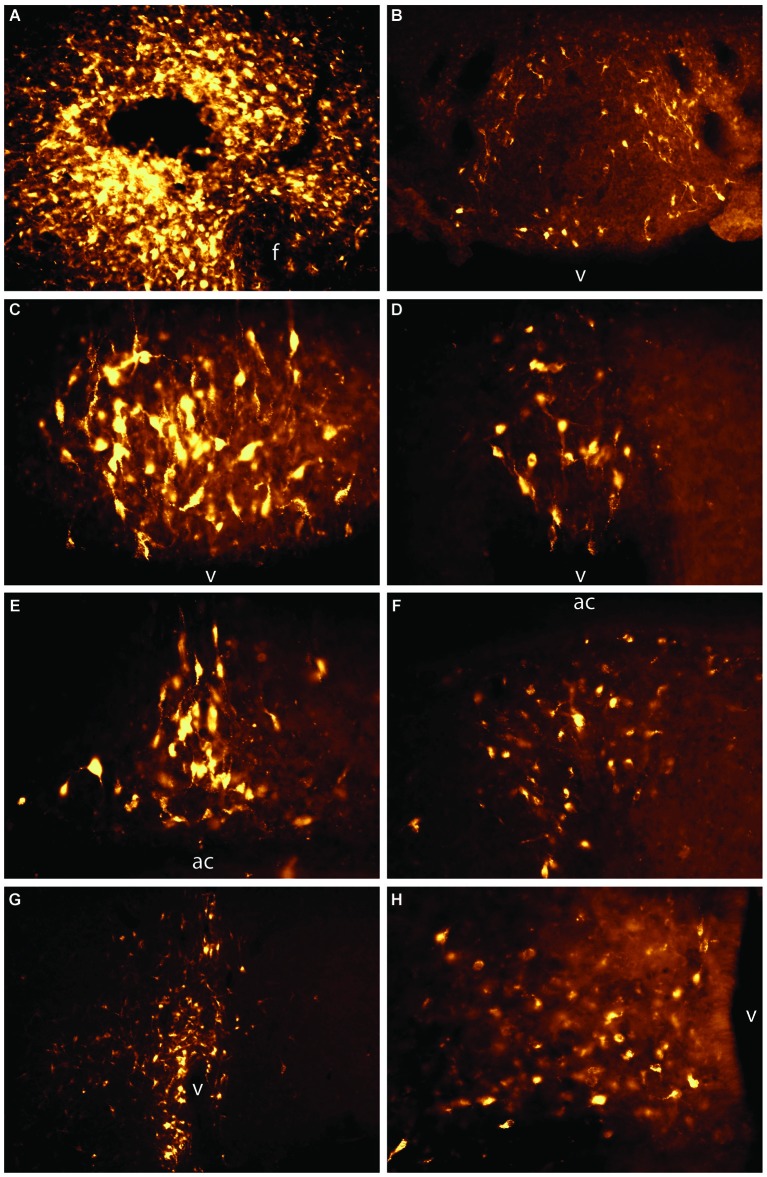
**Unpublished data from authors**. Retrograde labeling from the LHAd and PeF to the LT and arcuate nucleus of the hypothalamus. 2% Fluoro-Gold in physiological saline was iontophoresed into the PeF and LHAd **(A)**. Retrograde labeling was observed across the LT and in the ARH. Specifically, significant retrograde-labeling was seen in the annulus of the SFO **(B)**, anterior SFO **(C)**, stalk of the SFO **(D)**, dorsal and ventral MnPO **(E, F)**, OVLT **(G)**, and ARH **(H)**. Images were taken at different magnifications to compensate for the size of the brain area. Panels **(A)**, **(B)**, and **(G)** were taken at 10x and **(C)**, **(D)**, **(E)**, **(F)**, and **(H)** were taken at 20x. f = fornix, v = ventricle, ac = anterior commissure.

Anatomical and immunohistochemical studies support the idea that the LHA aids in integrating signaling from orexigenic peptides with neurocircuitry involved in motivation and reward. Neuropeptide Y (NPY) is expressed in ARH neurons (Hahn et al., 1998) and this neuropeptide induces feeding (Schwartz et al., 2000). Interestingly, NPY neurons send dense projections that are in apposition with orexin neurons located in the LHA (Broberger et al., 1998). Treatments that induce hunger such as hypoglycemia or administration of orexigenic peptides including ghrelin and NPY induce c-*fos* expression in orexin-containing neurons (Moriguchi et al., 1999; Niimi et al., 2001; Toshinai et al., 2003). Additionally, compromising orexin neurotransmission attenuates feeding induced by administration of either NPY or ghrelin. Neurons in the dorsomedial ARH, a region of the ARH that contains a majority of NPY neurons, also project to the PeF and possibly the LHAd (Figure 2; Hahn and Swanson, 2010).

In contrast to studies on food intake, relatively little work has been done delineating how the LT may influence motivation and reward neural circuitry. The LT does not appear to directly project to either the VTA (Phillipson, 1979; Geisler and Zahm, 2005) or the nucleus accumbens (Brog et al., 1993), but somehow areas sensing and processing information related to body fluid homeostasis must tap into motivation and reward neurocircuitry. The SFO has been shown to send projections to the DMH, PeF, and LHAd (Swanson and Lind, 1986; Hurley et al., 2013a). Furthermore, recent experiments in our laboratory have shown that iontophoretic application of the retrograde tracer Fluoro-Gold into the posterior portion of the DMH, PeF, and LHAd reveals retrograde labeling across the entirety of the LT (an example of retrograde labeling from an injection that spread from the PeF to the LH is presented in Figure 2). Others have shown that the PeF receives projections from the entirety of the LT (Hahn and Swanson, 2010). Additionally, we have recently found that orexin neurons are activated when rats depleted of water and sodium are allowed to ingest water and hypertonic saline and that microinjection of an orexin receptor antagonist into the VTA attenuated combined water and sodium intake in depleted rats (Hurley et al., 2013a). Therefore, it is likely that the LT projects to the DMH, PeF and LHAd which, in turn, send orexinergic projections to the VTA. Orexin release in the VTA promotes the ingestion of water and sodium. These experiments provide both anatomical and functional support to the hypothesis that the LHA integrates information about homeostatic state with motivation and reward systems.

## The evidence supporting a role for the LHA in reward-related learning

Experiments that examined the effect of sustained LHA stimulation on motivated behaviors provided some of the earliest evidence that the LHA may be involved in reward-related learning. When individual rats receive LHA stimulation they initially exhibit one specific motivated behavior (Valenstein et al., 1970). Some rats will eat, while others will drink or engage in copulatory behaviors. The motivated behavior each rat engages in is referred to as the prepotent behavior. Importantly, the prepotent behavior performed during LHA stimulation can be modified by experience (Valenstein et al., 1970). If the preferred goal object is removed during LHA stimulation, rats will direct their motivated behavior towards another goal object that is present in the environment. For example, if a rat eats during LHA stimulation, food can be removed while a drinking spout remains. In this situation the stimulated rat will now drink from the spout. Importantly, when the LHA is stimulated in future trials when both food and water are present, the rat will essentially split its time between eating and drinking. Therefore, pairing LHA stimulation with the presence of an initially non-preferred goal object causes a rat to direct some of its behavior toward the previously ignored goal object. It appears that LHA stimulation and the subsequent consumption of a goal object results in a form of associative learning that is expressed through changes in prepotent behaviors.

Orexin neuron activation can also become conditioned to stimuli in the environment. In conditioned place preference paradigms a novel environmental context is associated with a reward. After repeated pairings of an environmental context with a reward, rats will exhibit a preference for the context that was paired with reward. The preference that develops in conditioned place preference paradigms appears to be associated with orexin neuron activation. Orexin neurons express c-*fos* in response to environmental contexts that have become associated with drugs of abuse and sex (Harris et al., 2005; Di Sebastiano et al., 2011). Furthermore, lesioning orexin neurons with an orexin conjugated-saporin prevents male rats from exhibiting a conditioned place preference for an environmental context associated with copulation (Di Sebastiano et al., 2011).

Further evidence supporting LHA involvement in associative forms of reward learning comes from the phenomenon of cue-induced feeding. In the cue-induced feeding paradigm, food-deprived rats are allowed to eat in the presence of an environmental cue. This cue essentially becomes a conditioned stimulus (CS+) that is capable of inducing food intake. When the CS+ is presented to rats, even when they are in a satiated state, they will begin eating (Petrovich et al., 2007). Interestingly, rats will only ingest significant quantities of the specific food paired with the CS+, but not novel or familiar foods (Petrovich and Gallagher, 2007). Therefore, it appears that presentation of the CS+ evokes a specific craving for the food paired with the CS+, rather than hunger *per se*. The LHA is one area that is critical for the performance of cue-induced feeding (Petrovich and Gallagher, 2007; Petrovich et al., 2005). The LHA receives inputs from areas involved in associative forms of reward-learning including the amygdala (Krettek and Price, 1978; Everitt et al., 1999) and prefrontal cortex (Gallagher et al., 1999). Neurons that project to the LHA from the basolateral/basomedial amygdala and orbitomedial prefrontal cortex are activated in response to presentation of the CS+ (Petrovich et al., 2005). In addition, contralateral asymmetrical lesions of the basolateral amygdala and LHA prevent cue-induced feeding (Petrovich et al., 2005). Orexin also appears to plays a role in cue-induced feeding as rats exposed to the CS+ express significantly more c-*fos* positive orexin neurons in the PeF (Petrovich et al., 2012).

Finally, the LHA has been implicated in non-associative forms of reward-related learning. When rats are repeatedly depleted of sodium they exhibit an increase in sodium intake (Falk, 1965; Sakai et al., 1987, 1989), a phenomenon termed the sensitization of sodium appetite (Hurley et al., 2013b). Sensitization of sodium appetite is likely to be a form of non-associative learning (Falk, 1966; Frankmann et al., 1986) that is dependent on glutamatergic NMDA receptor-dependent neural plasticity (Hurley and Johnson, 2013). Evidence suggests that sodium appetite sensitization involves neural plasticity in two neural circuits: one circuit governing body fluid homeostasis and another circuit mediating motivation and reward (Roitman et al., 2002; Na et al., 2007). c-*fos* expression induced by sodium depletion is elevated in rats with a history of sodium depletions in the SFO, basolateral amygdala, medial prefrontal cortex, and nucleus accumbens compared to rats with no history of sodium depletions (Na et al., 2007). Furthermore, rats with a history of sodium depletions exhibit enhanced dendritic arborization and length in the nucleus accumbens (Roitman et al., 2002). Many of the areas that appear to undergo sensitization during sodium depletion also send projections to the LHA, including the SFO, prefrontal cortex, and basolateral amygdala. The LHA sends projections to the VTA, which in turn is capable of inducing neural plasticity in nucleus accumbens neurons (Mameli et al., 2009). Finally, additional evidence supports the possibility that orexin neurons undergo neural plasticity from sodium depletion (Liedtke et al., 2011). Activity-regulated cytoskeleton-associated protein, which plays a critical role in neural plasticity (Tzingounis and Nicoll, 2006; Shepherd and Bear, 2011), is upregulated in PeF orexin neurons during sodium depletion (Liedtke et al., 2011).

## Synthesis and conclusions

**Figure 3:**
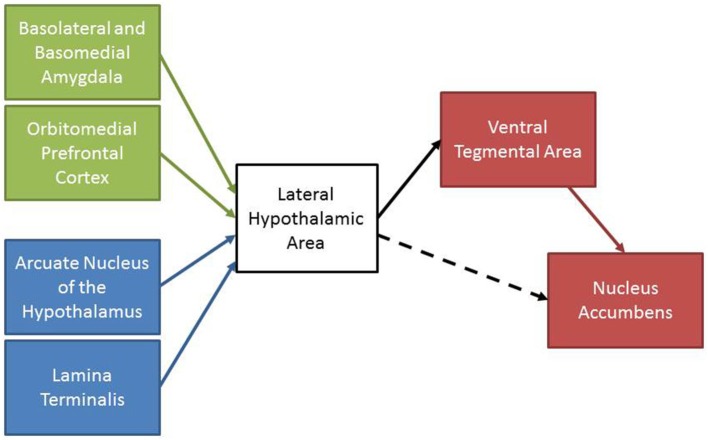
**A schematic summary of the reviewed experiments**. Areas involved in associative learning (green) and maintaining homeostasis (blue) project to the LHA. The LHA sends projections to motivation and reward areas (red) to initiate motivated behaviors. The LHA also sends projections to the nucleus accumbens that may promote motivated behaviors (dashed line).

The reviewed experiments support the hypothesis that the LHA contributes to the integration of information related to homeostatic state and past experience with motivation and reward systems. A summary of the anatomical and functional data is presented in Figure 3. Nuclei within the LHA, including the PeF and LHAd, receive projections from brain areas that regulate energy and body fluid homeostasis in addition to areas involved in associative learning (Broberger et al., 1998; Petrovich et al., 2005; Hurley et al., 2013a). In turn, these areas of the LHA send projections to the VTA where they promote motivated behaviors, at least partially through the release of orexin in the VTA (Phillipson, 1979; Fadel and Deutch, 2002; Geisler and Zahm, 2005). Although Figure 3 displays a hierarchical model of LHA functioning dominated by efferent connections to downstream brain areas, it may be the case that this circuitry is actually a neural network which consists of bidirectional inputs between areas involved in learning, homeostasis, and motivation and reward. In this respect, the use of anterograde and retrograde tracer co-injections would provide utility in identifying whether these areas form a neuronal network (for example see Thompson and Swanson, 2010).

As orexin is not exclusively involved in mediating just a single motivated behavior, it is likely that orexin acts to strengthen goal-directed responses associated with several motivated states (Borgland et al., 2009). Cues related to reward presentation and consumption can also induce activation of orexin neurons (Harris et al., 2005; Di Sebastiano et al., 2011; Petrovich et al., 2012), suggesting that past experience influences orexin neuron activity. Therefore, there are at least two conditions that induce orexin neuron activity: (1) the actual seeking and consumption of rewards; and (2) learned associations with rewards. With respect to the second point it is important to point out that orexin can induce neural plasticity in the VTA itself (Borgland et al., 2006). It is unlikely that orexin mediates all of the effects on motivated behaviors observed through manipulations of the LHA, as many projections from the hypothalamus to the VTA are non-orexinergic.

Future work that aims to carefully investigate the role of nuclei located within the LHA will prove fruitful. The LHA actually consists of a collection of heterogeneous brain areas that have unique neuroanatomical connections and cytoarchitecture (Swanson et al., 2005; Hahn and Swanson, 2010). Furthermore, it appears that separate orexin neuron clusters are activated under different experimental conditions (Harris et al., 2005; Harris and Aston-Jones, 2006; Petrovich et al., 2012). Optogenetic manipulations provide a method to test whether these orexin cell clusters have a functionally significant projection to the VTA or nucleus accumbens. Additionally, inactivation of orexin cell clusters should influence the activity of VTA and nucleus accumbens neurons. Finally, it is worth noting that many of the discussed experiments did not dissociate and define the roles of brain nuclei within the LHA and did not discuss the role of the DMH in motivated behaviors. The DMH also receives projections from body fluid homeostasis areas (Swanson and Lind, 1986), sends projections to the VTA (Geisler and Zahm, 2005), and contains orexin neurons (Fadel and Deutch, 2002), all of which are potentially involved in homeostatic behaviors.

## Health implications

From a behavioral perspective, some disorders can be conceived of as problems of ingestion. For example, anorexics fail to ingest sufficient amounts of food while those suffering from obesity ingest too much food. Similarly, some individuals ingest far too much sodium; a phenomenon sometimes referred to as salt gluttony (Schulkin, 1986), while others ingest too little sodium and consequently become sodium deficient causing them to experience autonomic and cardiovascular dysfunction (Bou-Holaigah et al., 1995). Additionally, elderly individuals may exhibit diminished thirst and subsequent dehydration (Rolls and Phillips, 1990; Warren et al., 1994). One approach to understanding these maladies that are marked by a surplus or surfeit in ingestive behavior is to conceive of them as problems of central nervous system functioning related to maintaining homeostasis and appropriately engaging in motivated behavior. As the LHA is critically involved in both maintaining homeostasis and mediating motivated behaviors, an improved understanding of the LHA may aid in diagnosing and treating disorders of ingestion.

## Conflict of interest statement

The authors declare that the research was conducted in the absence of any commercial or financial relationships that could be construed as a potential conflict of interest.
